# A 3D image-based modelling approach for understanding spatiotemporal processes in phosphorus fertiliser dissolution, soil buffering and uptake by plant roots

**DOI:** 10.1038/s41598-022-19047-1

**Published:** 2022-09-23

**Authors:** K. A. Williams, D. M. McKay Fletcher, C. Petroselli, S. A. Ruiz, T. Roose

**Affiliations:** 1 Faculty of Engineering and Physical Sciences, University of Southampton, Southampton, UK; 2 Faculty of Science and Health, University of Portsmouth, Portsmouth, UK

**Keywords:** Environmental impact, Element cycles, Computational models, Image processing

## Abstract

Phosphorus (P) is a key yield-limiting nutrient for crops, but the main source of P fertiliser is finite. Therefore, efficient fertilisation is crucial. Optimal P application requires understanding of the dynamic processes affecting P availability to plants, including fertiliser dissolution rate and soil buffer power. However, standard soil testing methods sample at fixed time points, preventing a mechanistic understanding of P uptake variability. We used image-based modelling to investigate the effects of fertiliser dissolution rate and soil buffer power on P uptake by wheat roots imaged using X-ray CT. We modelled uptake based on 1-day, 1-week, and 14-week dissolution of a fixed quantity of total P for two common soil buffer powers. We found rapid fertiliser dissolution increased short-term root uptake, but total uptake from 1-week matched 1-day dissolution. We quantified the large effects root system architecture had on P uptake, finding that there were trade-offs between total P uptake and uptake per unit root length, representing a carbon investment/phosphorus uptake balance. These results provide a starting point for predictive modelling of uptake from different P fertilisers in different soils. With the addition of further X-ray CT image datasets and a wider range of conditions, our simulation approach could be developed further for rapid trialling of fertiliser-soil combinations to inform field-scale trials or management.

## Introduction

Phosphorus (P) is a key limiting nutrient for plant growth^1,2^. To keep up with global food demand, phosphate fertiliser is applied to fields at a rate of nearly 50,000,000 tonnes each year, and the demand for P is growing at a rate of around 2.2% a year^3^. However, most of the global P fertiliser supply is mined from rock phosphate, a resource so limited that it could run out entirely in just over 250 years or sooner depending on how demand grows^4^. Despite this growing threat, modern agriculture often applies excessive fertiliser quantities with the goal of maximising short term yields^5^. Not only is this an inefficient use of the increasingly-scarce resource, it can also lead to a saturation of P in the soil which can cause P run-off and leaching into water sources with serious environmental consequences. To ensure sustainable use of P in agriculture, strategies are required to decrease unnecessary P fertilisation and increase plant uptake from the fertiliser (fertiliser use efficiency) while maintaining high yields and nutritious crops^5,6^.

Approaches to increase fertiliser efficiency typically make improvements to at least one of the ‘4Rs’: the right nutrient source, the right rate of application, the right time of application, and the right place^7^. This requires an understanding of how different fertilisers respond to different soils, how soluble they are, and what that means for its availability to plants. While conventional experimental approaches can test overall efficacy of a particular application on final crop yields, they provide limited insight into the dynamic processes that influence how effective a particular fertiliser in a particular environment can be^8^. An understanding of the early dynamics of P availability is particularly important given yield is known to depend on early P uptake^9^.

The availability of P to plants is largely determined by the buffering of phosphates to soil constituents; this is high due to the low solubility of P minerals and fast adsorption of P onto soil particles. This reduces the distance that P moves in the soil and ultimately limits the uptake of P by plant roots^1,10^. Thus, the P uptake from soil and added fertilisers is often inefficient^1^. In the context of fertilisation, P availability is also affected by the solubility of the fertiliser^9^. Some fertilisers such as triple-super phosphate (TSP) dissolve rapidly in water^11^, while others, such as struvite, dissolve much more slowly, as only 30% of the mass of struvite pellets dissolved after a 14 week experiment in soil^12^. Solubility in water is not necessarily indicative of how effective a fertiliser will be in an agricultural context since typical agricultural soils are often unsaturated and contain a complex mixture of biological and abiotic components that affect dissolution rate and dispersal^13^.

Due to the numerous processes affecting P availability it is not clear if faster fertiliser dissolution necessarily results in higher plant uptake. For example, a highly soluble fertiliser could release its P before roots are within range and the P could become immobilized to soil particles before the plant has the chance to use it^14^. Nkebiwe et al.^15^ found that fertiliser sources with fast dissolution had greater yield and nutrient uptake compared to slow dissolution fertilisers. In contrast, other researchers^9^ found fertiliser recovery was similar between diammonium phosphate (DAP), which is a relatively soluble source, and struvite for spring wheat at harvest.

Precision application of fertilisers requires an understanding of dissolution rates, sorption onto soil, plant availability, and potential run-off. Fertilisers with different dissolution rates aim to target either rapid release for immediate plant uptake or sustained release, with some claiming to only release fertiliser in the presence of plant roots. There is a current focus on alternative P sources from recycled materials and methods to increase P availability to plants that include coating soluble pellets^9,16^. These slow release fertilisers have potential benefits in reducing runoff^17^, and some are considered more sustainable due to their recycled sources. However, the efficacy of these different fertilisers varies between studies, with some indicating that slow release fertilisers can be highly effective^18,19^, and others suggesting that they are insufficient^20^. Some of these differences are likely due to experimental designs^21^, soil variability, and variation in root architectures^22^. Additionally, plants have different nutrient requirements at different stages of growth. They need a large amount of P early in growth, so a P fertiliser that dissolves more slowly may not provide enough P at the critical time and may be less effective in terms of increased yield^9,20,23^. Finally, P that is bound to soil may resolubilise and become available again. However, this is seldom considered in the context of agricultural systems^24^. Therefore, the trade-offs between rapid release vs sustained release are not fully understood.

To rigorously assess the function of different fertilisers applied at different stages in plant development, it is important to understand how the P movement and availability evolves over time. However, standard soil field protocols often only provide snapshots and can lack the capacity to disentangle small-scale biogeochemical processes and proximity of roots to fertilisers. Recent studies have begun to focus on these processes at greater spatial and temporal resolution using soil solution sampling techniques such as suction cups^25^ and microdialysis sensors^8,11,26^. These studies have provided new insights into the P dynamics in soil and highlighted the need to revisit and update our preconceptions of these processes at fundamentally relevant scales.

While experimental approaches can provide some spatial and temporal resolution, modelling approaches can fill in measurement gaps and allow us to test different scenarios more rapidly. This could provide a complementary approach to focus and enhance more costly and time consuming field trials. Image-based modelling (using experimental images to define the geometry of models) offers vital advantages over models where root structure are represented by parameters or functions for the problem of assessing fertiliser dissolution rates and P availability and can complement results from imaging studies^27^. When using the latter modelling approach, root geometry including proximity to high P sources is averaged and thus important processes for fertiliser efficiency are overlooked. In particular, the details of root competition for the same soil/fertiliser P supply within a discretised area/length are averaged without consideration for localised root interactions^27^. With image-based modelling, not only is explicit root fertiliser proximity calculated from experimental data, but competition between roots (of the same plant) for local P supplies are simulated^22^, allowing accurate assessment of P uptake efficiency, in the sense of unit of P absorbed per unit of photosynthate spent of root growth, from fertilisers. Including such geometric detail in image-based models comes at large computational cost. Thus, image-based models are less suitable for research questions where multiple-physics and/or larger spatial scales need to be considered, and models that represent roots by parameters or functions are the preferred modelling approach^27^.

This study investigated the fundamental processes governing the efficacy of different P fertiliser dissolution rates and developed a protocol that could be used to test a range of different fertiliser and soil conditions, which can be used to predict efficient fertiliser applications in space, time and quantity given soil and fertiliser properties. To this aim, we developed an image-based modelling approach to investigate how P dissolution rates and soil buffering affect P availability to plant roots. We used X-ray CT data from a previous study^12^ and compared anatomically the root systems of spring wheat plants treated with a fertiliser pellet. We then assessed P uptake from the fertiliser pellet using image-based modelling assuming a range of model fertiliser dissolution rates (similar to TSP or struvite) and soil P sorption properties. We aimed to quantify the effect of fertiliser dissolution on plant uptake of P from the pellet, and investigate the role of root system architecture and pellet location in these processes. We expected the benefit of the fertiliser pellet to be more noticeable in the high buffering soil due to lower available P in the bulk soil. We also expected that there would be more variability in P uptake in the high buffering soil because low P mobility would cause greater local variation in P concentrations.

## Methods

### Experimental setup

The experimental system for X-ray CT imaging^12^ was a PVC column with a diameter of 110 mm and height of 500 mm. The column was filled with a P-deficient (Olsen P = 12.6 mg l^−1^) sandy loam textured Eutric Cambisol taken from depths between 0–15 cm depth (Ahp horizon) collected from a freely draining grassland located in Abergwyngregyn, UK (53°14′ N, 4°01′ W) and sieved to pass 5 mm. The columns were sufficiently large to allow the plants to grow to maturity. The cylindrical shape and relatively narrow diameter facilitated XCT imaging. Single spring wheat plants (*Triticum aestivum* L. cv Granary; KWS UK Ltd., Thriplow Hertfordshire, UK) were grown in each column. See Ahmed, et al.^12^ for full experimental setup including water, light, and actual fertiliser conditions. Three replicates were analysed.

### XCT imaging

Columns were imaged using a custom-design micro-focus Nikon ‘hutch’ μCT scanner (Nikon Metrology Europe, Leuven, Belgium) at 14 weeks post-planting. For each column, image data were acquired using an energy of 150 kV, and a power of 15.15 W. 3142 projections were collected over 360° rotation using a flat panel detector with 2048 × 2048 pixels, and the voxel size was 60 μm. The average scan time for each column was 56 min. Reconstructions were carried out using a proprietary filtered back-projection algorithm implemented in CTPro 3D (Nikon Metrology, Tring, UK).

### Image processing

Roots were segmented from the surrounding soil using a custom workflow, with a combination of non-local means filtering, grey value thresholds, morphological filters, and shape-based filters implemented in a combination of the FIJI distribution of ImageJ^28,29^, a free, open-source software package for image processing and analysis, and Dragonfly (ORS, Quebec, Canada), a commercial software package for image processing, analysis, and visualisation (Figs. S1 and S2). Exact values used for these datasets for variables such as greyscale thresholds are provided but may need to be adjusted for other experiments where soils or X-ray settings differ. Image stacks were first converted to 8bit using a fixed grey value range (− 20 to 80 in the 32 bit range which was found to include most of the data without overexposing the brighter regions) in order to reduce computational time. A non-local means filter^30^ was then applied, using a damping parameter *h* = 0.106, a half window size *B* = 2, and a search window size *W* = 5 which removed much of the image noise while preserving edges of roots. After filtering, the soil particles were segmented from the background using a fixed greyscale threshold of 107, where values above 107 are ‘soil’ and below 107 are ‘not soil’. Each soil particle was surrounded by a region of pixels with grey values that were lower than the soil but higher than the air, due to partial volume effects. This region happens to coincide closely with the grey values associated with roots and it was therefore important to remove these voxels. Therefore, a 2-pixel 3D dilation, implemented in the MorphoLibJ plugin for ImageJ^31^ was used to include those pixels within the regions classified as ‘soil’. The ‘soil’ regions were then removed from the original greyscale image in order to segment the roots. The roots were over segmented from the greyscale ‘not soil’ image using a second fixed grey value threshold of 89, where values above 89 were classified as ‘root’ and values below 89 were classified as ‘not root’. A 1 pixel 3D opening function was used to remove small elements of noise, followed by a 2 pixel 3D closing function to connect small gaps in the root system. Following this process, the binarised image was saved as a stack of TIFFs then imported into Dragonfly. In Dragonfly, a connected components analysis identified parts of the image that were connected and labelled them. The number of voxels and the aspect ratios of each labelled object were measured. The largest parts of the root systems were selected manually, while smaller and disconnected roots were identified as those objects that were greater than 1000 voxels and had an aspect ratio of less than 0.05 (i.e. were long and thin). Due to imaging constraints (60 µm resolution) and processing throughput (1 pixel opening operation), any roots smaller than 180 µm will not be visible in the image.

### Root measurements

Segmented root systems were measured using the BoneJ plugin of ImageJ^32^ (Fig. S3). Each root system was skeletonised (thinned to a single line of pixels) using the ‘Skeletonize 2D/3D’ tool. In thicker parts of the segmented roots, artificial loops formed in the skeleton (an artefact which would lead to significant over measurement of length density). Thus, the skeletons were cleaned of artificial loops by filling the holes (i.e. filling the loops) in 2D and reskeletonising so that the loop was now a single central pixel (Fig. S3). The root length density was measured by counting the number of white (root skeleton) pixels in each slice and dividing by the volume of soil in the slice to give a measure of root length density in mm of root per cm^3^ soil.

Root length density with distance from pellet was measured to aide interpretation of the modelling results. The exact positions of the fertiliser pellets were difficult to assess since they disintegrated over time. However, their relative depth from the surface was measured from images taken at the start of the experiment where at least one pellet could be seen clearly for each treatment. All pellets were approximately 240 mm (400 XCT slices) from the soil surface and therefore this depth was chosen to represent the fertiliser pellet depth. The exact radial positions of each pellet were unknown, but P sources representing the pellets were added in silico as detailed below in the modelling section. This ‘pellet’ was then used as the basis for a distance transform using ‘Exact Distance Transform 3D’ in ImageJ (Fig. S4). Greyscale thresholds were applied to segment the distance transform at intervals of 2 mm with respect to the pellet (see Supplementary Material). The pixels in the skeleton image were then counted in each of the distance regions and this was used to calculate a root length per cm^3^. Root diameters were measured using the “Thickness” tool within BoneJ which uses a sphere fitting approach to calculate mean diameter of structures.

To measure the mean distance from the pellet to the roots, the skeleton of the roots was multiplied with the distance map of the pellet, and the mean grey value calculated across the stack. Lastly, a proxy for the extent that a root system architecture had explored was assessed by computing the mean distance between soil (i.e. each voxel within the soil domain) and its nearest root. This we refer to as mean soil–root distance and is calculated using the mean grey value of a distance transform from the roots (Fig. S5).

### Image based modelling

**Figure 1:**
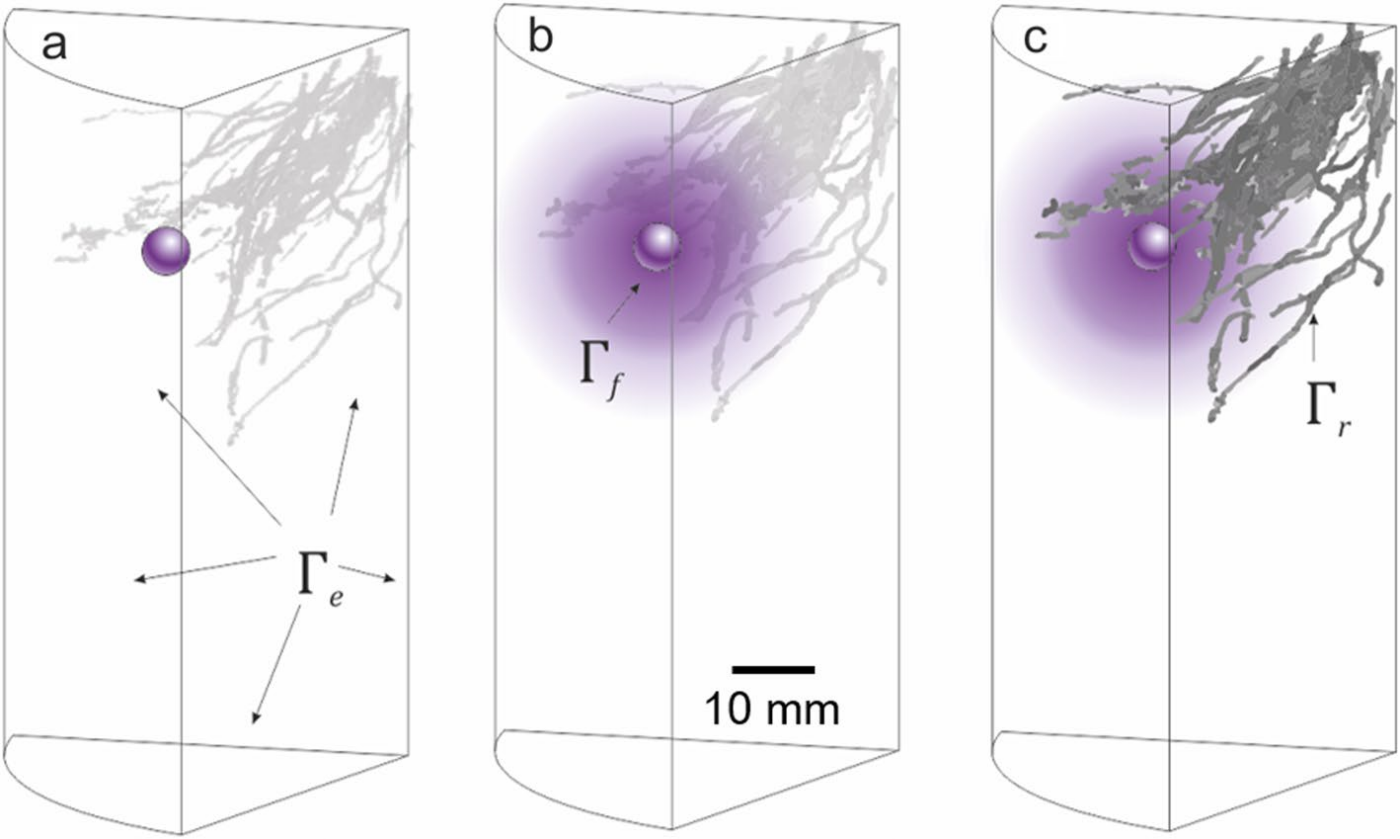
Visual description of 3D image-based modelling domain and model progression. The surface boundaries representing the edge of the pot and internal artificial boundaries where we subsample the image, ${\Gamma }_{{\text{e}}}$ are shown on (**a**); the fertiliser surface, ${\Gamma }_{{\text{f}}}$ on (**b**); and the root surfaces, ${\Gamma }_{{\text{r}}}$ on (**c**). (**a**) The initial state of the model with the roots deactivated and the fertiliser pellet primed to start dissolution; (**b**) In the first 6 weeks the roots remain inactive but the fertiliser pellet releases phosphorus into the soil which diffuses over this period; (**c**) After 6 weeks the roots reached the pellet (as calculated from the time-resolved XCT-data) and the roots are activated and take-up phosphorus from the soil. The phosphorus continues to diffuse in the soil. Purple shading is for illustrative purposes only.

We developed an image-based model to allow us to better resolve spatial and temporal transport of P fertiliser. The model enabled inferences for unmeasured quantities in the study such as P concentrations in the soil and root P uptake. Furthermore, the modelled system was used to simulate different fertiliser solubilities for different root system architectures. The classical Nye–Tinker–Barber model was used to represent P movement in a 3D homogeneous-soil domain containing 14-week root systems^1^. The roots and fertiliser pellets were treated as surfaces extracted from the XCT images in the soil domain (shown in Fig. 1), with Michaelis–Menten kinetics as the root boundary condition to represent P uptake^1^ and decaying P flux as the fertiliser boundary condition to represent fertiliser dissolution. More precisely, let ${\Omega } \subset {\mathbb{R}}^{3}$ represent the soil in the column. We assume the soil is homogenous with constant volumetric water content $\phi_{l} = 0.3$ [m^3^_soil liquid_ m^−3^_bulk_] and constant volumetric soil solid content $\phi_{s} = 0.6$ [m^3^_soil solid_ m^−3^_bulk_] to be consistent with the model of McKay Fletcher et al.^8^, however, it is likely the water content varied in the experiment. The boundary of the soil consists of the edge of the column, ${\Gamma }_{e}$, the root surfaces, ${\Gamma }_{r} ,$ and the fertiliser surfaces ${\Gamma }_{f}$ which are shown in Fig. 1. Due to the small Péclet number for P in soil^33^, movement of P in the soil is assumed to be governed by diffusion only. The linear adsorption–desorption reaction of P onto soil surfaces is assumed to be in equilibrium so adsorbed P can be approximated by P in solution using the buffer power, *b*, the ratio of sorption and desorption rates^1^. Let $P_{l} ({\varvec{x}},\;t)$ [µmol m^−3^_soil liquid_] represent the concentration of P in soil solution, then the movement of P in homogeneous soil is governed by the equation

1 $$\phi_{l} \left( {1 + b} \right)\frac{{\partial P_{l} }}{\partial t} = \nabla \cdot \left( {D\phi_{l} f\nabla P_{l} } \right),\quad {\varvec{x}} \in {\Omega },$$

where *b* = 40 (low buffering soil^8^) or 519 (high buffering soil^11^) [–] is the buffer power of *P*_*l*_ in this soil, defined as $b = \frac{{k_{1} }}{{k_{2} }}$, where *k*_1_ [s^−1^] and *k*_2_ [s^−1^] are the adsorption desorption rates in the first-order kinetics describing soil sorption respectively. $D = 7 \times 10^{ - 10}$ [m^2^ s^−1^] is diffusion coefficient of P in soil solution and *f* = 0.3 [–] is the geometric impedance to diffusion from the soil structure^1^. At the edge of the soil column we impose a no-flux boundary condition so P cannot leave the domain

2 $${\varvec{n}} \cdot \left( {D\phi_{l} f\nabla P_{l} } \right) = 0,\quad {\varvec{x}} \in {\Gamma }_{e} ,$$

where is ***n*** is the outward pointing unit normal to the surface.

On the root surfaces we impose Michaelis–Menten kinetics as the boundary condition representing the absorption of P by the roots. Since the 14 week root surfaces are stationary (i.e. root growth is not modelled) we use a step function to activate the roots once they have reached the fertiliser pellet depth,

3 $${\varvec{n}} \cdot \left( {D\phi_{l} f\nabla P_{l} } \right) = \frac{{ - F_{P} P_{l} }}{{K_{P} + P_{l} }}H\left( {t - t^{*} } \right),\quad {\varvec{x}} \in {\Gamma }_{r} .$$

where $F_{P} = 3.26 \times 10^{ - 2}$ [µmol m^−2^ s^−1^] is the maximum possible root P uptake rate, $K_{P} = 5.8 \times 10^{3}$ [µmol m^−3^] is P concentration when the root uptake rate is half $F_{P}$^1^ and $t^{*} \left[ s \right]$ is the time the roots reach the depth of the pellet as calculated from the X-ray CT scans (6 weeks). Since only the 14 week root system is used as the geometry for the model, the Heaviside function, $H\left( t \right)$, is used to activate root uptake at $t^{*}$. With this representation of root uptake, model results only measure P uptake from 6 to 14 weeks, see Fig. 1 for a visual description of root activation. A fertiliser pellet is assumed to release its entire P content ($P_{fert} = 450$ µmol, determined experimentally) either over the 14-week simulation (representing a slow-release pellet such as struvite), 1-week or 1-day (fast release pellet such as TSP) with the release rate decaying exponentially in time^34^,

4 $${\varvec{n}} \cdot \left( {D\phi_{l} f\nabla P_{l} } \right) = J_{0} {\text{exp}}\left( { - t/\tau } \right),\quad {\varvec{x}} \in {\Gamma }_{f} .$$

where $J_{0}$ [mol m^−2^ s^−1^] is the initial release rate (at $t = 0$) of P from the pellet and $\tau = 14$ weeks, 1 week or1 day. $J_{0}$ is calculated to match the total P flux from a fertiliser pellet over $\tau$ days for all treatments. The total flux is set to $P_{fert}$ and by assuming each pellet to be a sphere with radius of 2 mm with equal P flux from each surface this results in flux expression $J_{0} = \frac{{P_{fert} }}{{A\tau \left( {1 - e^{ - 1} } \right)}}$, where *A* is the surface area of the 2 mm sphere representing the fertiliser pellet. The initial concentration of P in soil solution is assumed to be uniform in the soil and is chosen depending on the buffer power of the soil so that the total soil P is same between different simulations. For the low buffering case ($b = 40$) the initial P in solution concentration is chosen as $P_{l} \left( {{\varvec{x}},\;t = 0} \right) = P_{0}$ where $P_{0} = 2.53 \times 10^{5}$ µmol m^−3^ soil solution as calculated from another study using the same soil^35^. To ensure the same total amount of P in the soil between the low and high buffering case, when $b = 519$ the initial P concentration in the high buffering soil is 0.02 μmol m^−3^, calculated as

5 $$P_{l} \left( {{\varvec{x}},\;t = 0} \right) = \frac{{P_{0} \left( {1 + b_{l} } \right)}}{{1 + b_{h} }}{ }$$

where $P_{0}$ is the initial condition in the low buffering soil, $b_{l} = 40$ is low buffer power and $b_{h} = 519$ is the high buffer power.

### Meshing and numerical experiments

As the root systems extracted from the X-ray CT scans were very large, simulations using the full root system geometry could not be obtained due to computer memory and processing limitations. Instead, each column was divided into 6 sectors each running the full length of the image stack and one sector was chosen at random to be used for simulations, as each sector is expected to contain 1 fertiliser pellet. This process introduces an artificial boundary to ${\Omega }$ on which we impose a no-flux condition to obtain a symmetric solution in the whole domain, see Fig. 1. At 14-weeks the exact location of the fertiliser pellets could not be determined due to disintegration. Instead, a 2 mm radius sphere was added to the geometries at 24 mm below the soil surface and 25 mm from the centre of the column in the centre of the chosen column sector to represent the fertiliser pellet.

We hypothesised that faster fertiliser dissolution would improve plant P uptake from the pellet, but root system architecture would play a large role in fertiliser uptake efficiency. Additionally, we expected the benefit of the fertiliser pellet to be more noticeable in the high buffering soil due to lower available P and the role of root system architecture to be more prominent due to low P mobility. To assess these hypothesis, the modelled dynamic uptake rate was quantified for a number of scenarios. For each buffer power and root system, four cases were simulated: Complete fertiliser dissolution takes 14-weeks ($\tau = 14$ weeks), 1-week ($\tau = 1$ week), 1-day ($\tau = 1$ day) dissolution and Zero fertiliser $(\lim \tau \to 0)$ resulting in 24 simulations with 3 replicates for each dissolution regime and buffer power. Measurements of the model results include total P uptake over the 14 week simulation [µmol], uptake rate [µmol s^−1^] and P uptake efficiency, defined as either total uptake per root surface area [µmol m^−2^] or uptake rate per root surface area [µmol m^−2^ s^−1^].

The segmented roots, homogeneous soil and fertiliser pellet were then meshed using Simpleware ScanIP (Synopsys Inc, California, USA). The homogeneous-soil volume meshes typically had an element–volume ratio of $5.9 \times 10^{ - 6}$ average element quality of 0.7. The root and fertiliser surface mesh typically had element–area ratio of $9.8 \times 10^{ - 4}$ with an average element quality of 0.72. The simulations were solved in Comsol 5.5 (COMSOL Inc, Massachusetts, UK) using two bespoke computers with 528 GB of memory and 4 nodes each with 6 cores (AMD Opteron Processor 6344).

### Statistics

One way ANOVA was used to test for differences between means while two-sample t-tests were used for specific pairwise comparisons between means of model results. A threshold of p = 0.05 was chosen as significant. All statistics were calculated using scipy^36^.

### Use of plants statement

All local, national or international guidelines and legislation were adhered for the use of plants in this study.

## Results

### Imaged root length densities

**Figure 2:**
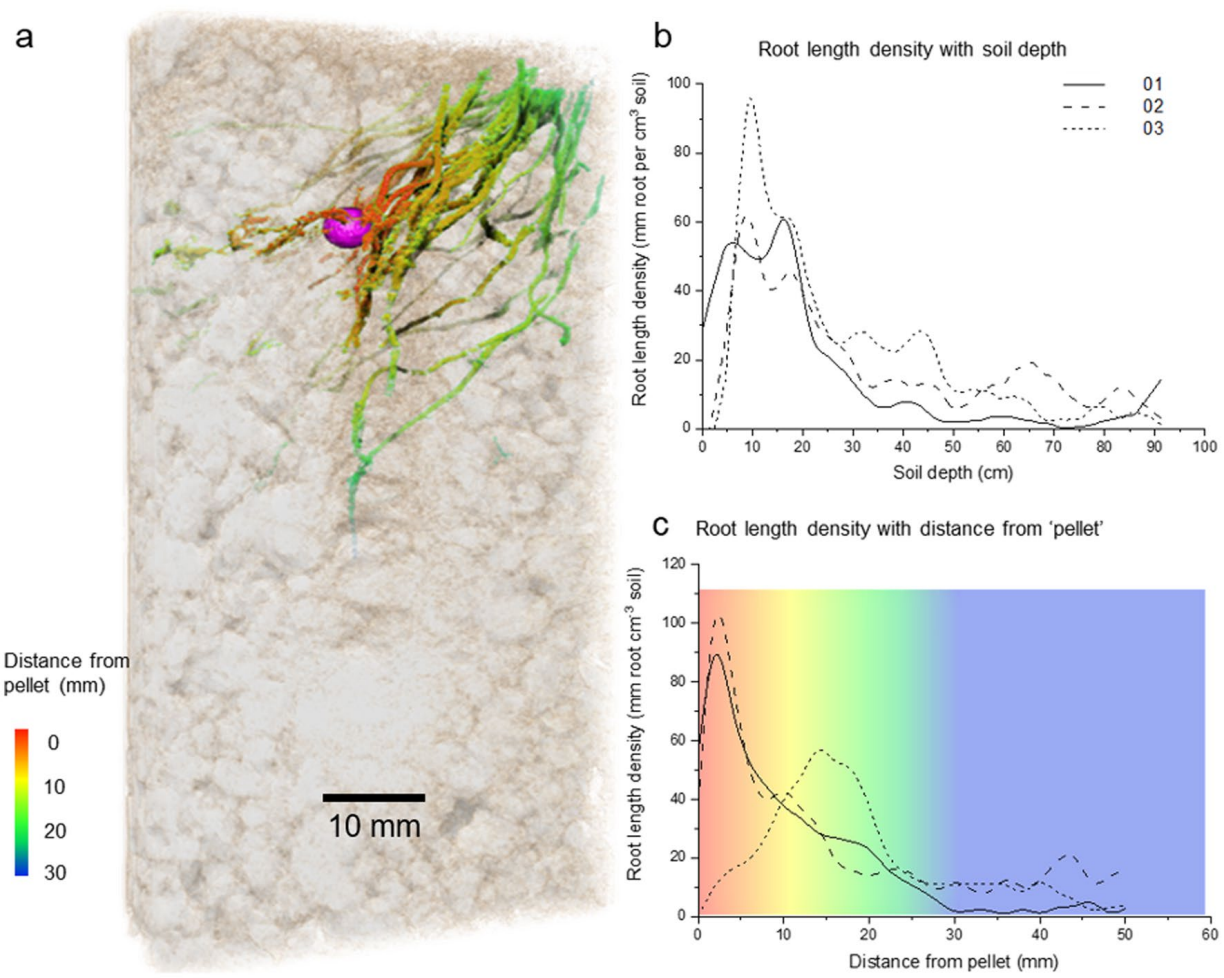
Visualization and root length density measurements in the randomised sectors. (**a**) 3D rendering of the sample 01 root system in the randomized sector. The approximated location of the fertiliser pellet is rendered in purple and the root are coloured by their distance from the fertiliser pellet. (**b**) Root length per depth. (**c**) Root length density with distance from estimated positions of fertiliser pellets (distances are maximum distances in the chosen range i.e. 1 cm = 0–1 cm) in 14 week old spring wheat plants.

To model the P uptake, the whole image stack was divided into six equal sectors using the full depth of the stack and a sector from each stack was selected randomly. For measurements, and for modelling going forwards, results refer to the selected sector only. A P source was placed within the sector, in a location consistent with the depth of the experimental pellet, Fig. 2a visualizes this root system 01 and each root system (01, 02 and 03) can be seen in Fig. S6 in the Supplementary Information. The changing root length density with depth and distance from the in silico P source was measured within the sector modelled. The pattern of root length density with depth is similar between replicates (Fig. 2b, 01, 02, 03). With distance from the ‘P source’, root length density generally decreased (Fig. 2b). It should be noted that this is relative to the in silico placement of the P source to give context for the model results rather than a measure of actual root responses. Two of the plants (01 and 02) had similar patterns of root length density with distance from the P source, while sample 03 had few roots within the first ~ 1 cm from the pellet, but a large number slightly further away at ~ 1.5 cm (Fig. 2c). Measurements of root surface area, how far each root surface is from the pellet and how far each soil volume is from a root can be found in Table 1. On average, sample 01’s roots are closest to the fertiliser pellet, followed by sample 03 then sample 02. However, sample 03 was the only treatment not to have roots immediately adjacent to the pellet, as indicated by the high minimum root-pellet distance. Although sample 02 had the lowest root surface area, its root system had explored more of the soil than any other treatment indicated by the lowest average soil–root distance (on average, how far is each piece of soil from its nearest root) (Table 1).

**Table 1:** Image analysis measurements of root surface area, average root distance from pellet and average soil distance from root in the sector selected for modelling. The maximum and minimum are also included to demonstrate the variation in the data.

	Root surface area (mm^2^)	Root–pellet distances (mm)			Soil–root distances (mm)		
		Mean	Min	Max	Mean	Min	Max
01	2888.6	31.14	0	74.84	8.54	0	27.54
02	1940.2	32.94	0.19	74.54	5.35	0	25.44
03	3724.2	32.04	1.78	67.44	7.97	0	27.44

### Modelled uptake trends

**Figure 3:**
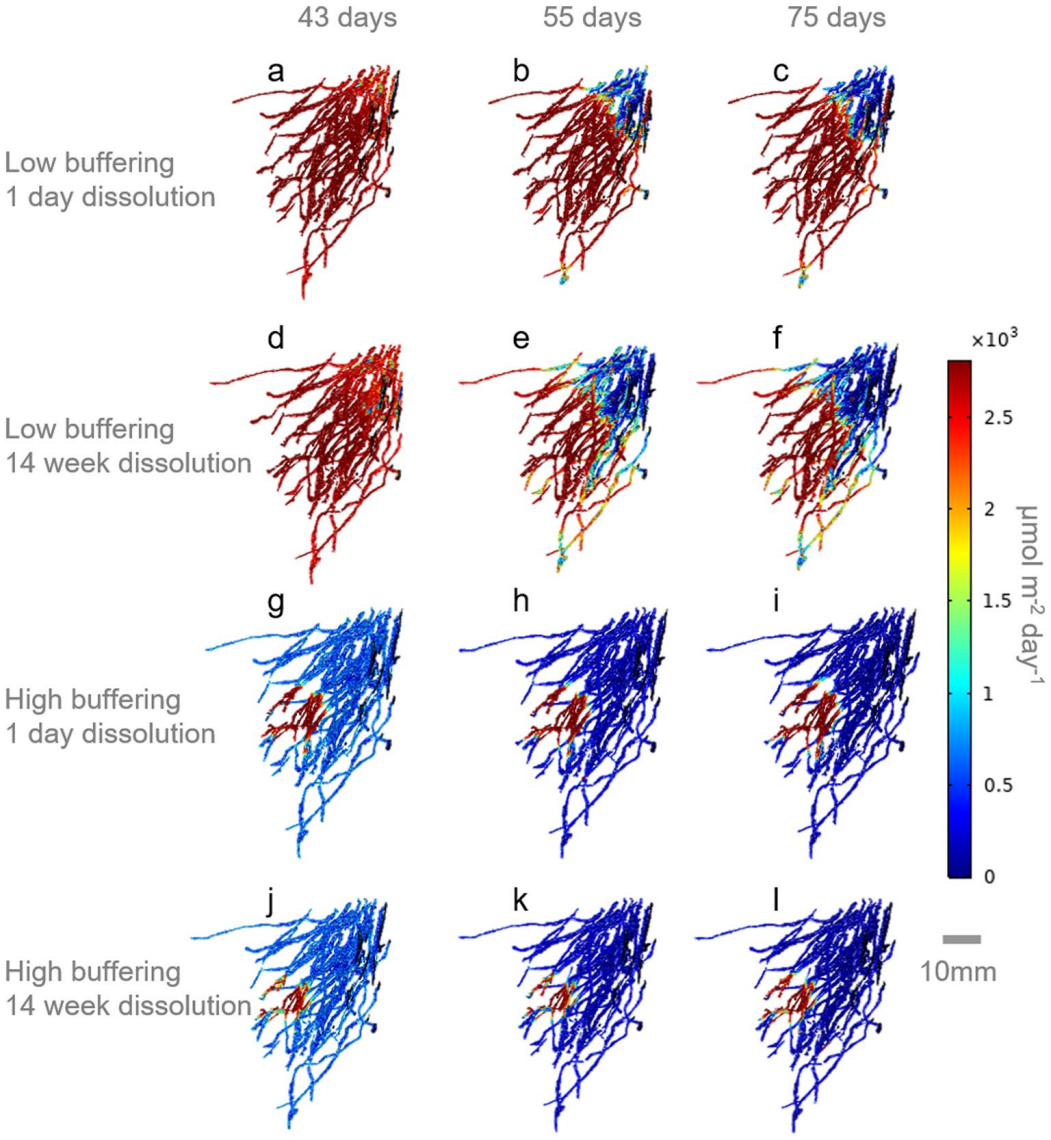
Visualisation of the root uptake rate evolution of the sample 01 root system for the 1-day and 14-week dissolution treatments. Pellet dissolution began at Day 0, root P uptake was switched on at Day 42. Day 43, 55 and 75 are selected for visualisation purposes. (**a**–**c**) Show the low buffering soil with 1-day fertiliser dissolution. (**d**–**f**) Show the low buffering soil with 14-week fertiliser dissolution. (**g**–**i**) Show the high buffering soil with 1-day fertiliser dissolution. (**j**–**l**) Show the high buffering soil with 14-week fertiliser dissolution.

In Fig. 3 the root uptake rates at select times are shown on the root geometry for the sample 01 root system. A large portion of the roots system is affected by the pellet in the low buffering soil (Fig. 3a–f) compared to the high buffering soil (Fig. 3g–l). In the high buffering soil, root dense regions further from the pellet seem to drop in uptake rapidly as P is taken up from the soil.

**Figure 4:**
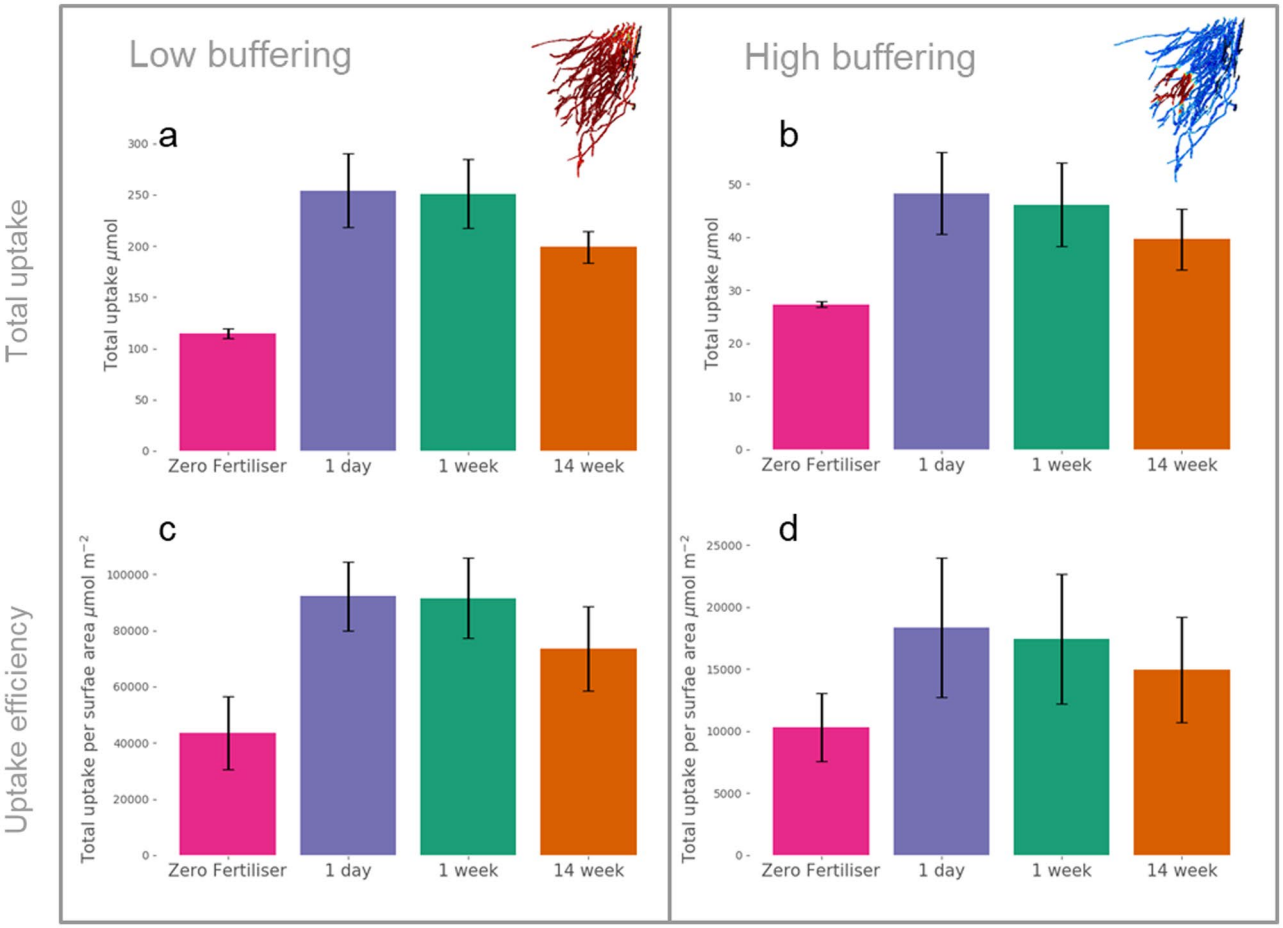
Simulated total phosphorus uptakes integrated over the whole experiment for different P dissolution rates averaged across 3 root systems (**a**,**b**) and total uptakes per root surface area (**c**,**d**) over the fertiliser treatments. Error bars show standard deviation, n = 3. (**a**) Total uptake averages in the low buffering soil. (**b**) Total uptake averages in the high buffering soil. (**c**) Total uptake per root surface area averages in the low buffering soil. (**d**) Total uptake per root surface area averages in the high buffering soil. For visualisation purposes, root images show uptake rate at 43 days.

In terms of total P uptakes over the 14-week simulation, there was no significant difference in either absolute P uptake or P uptake efficiency between the different fertiliser dissolution rates (one way ANOVA) (Fig. 4). However, any application of fertiliser significantly increased both P uptake and uptake efficiency in the low buffering soil (Student’s *t* test, two tailed, p = 0.0007 and 0.004 respectively) and only P uptake in the high buffering soil (p = 0.0067) compared to the zero fertiliser treatment. By adding the fertiliser, the total uptake was improved by 104% in the low buffering soil and 63% in the high buffering soil on average. Introducing fertiliser also increased the variability in total uptake, but to a lesser extent the variability in uptake efficiency between the root systems compared to the zero fertiliser treatment, Fig. 4. Additionally, the mean-normalised standard deviations of total uptakes in the high buffering soil were each greater than the low buffering soil in all fertilisation treatments, but lower in the zero fertiliser treatment. These higher variations amongst root systems are consistent with our hypothesis that root system architecture is more important feature in high buffering soils than lower buffering soils for P fertilisation.

### Modelled uptake dynamics

**Figure 5:**
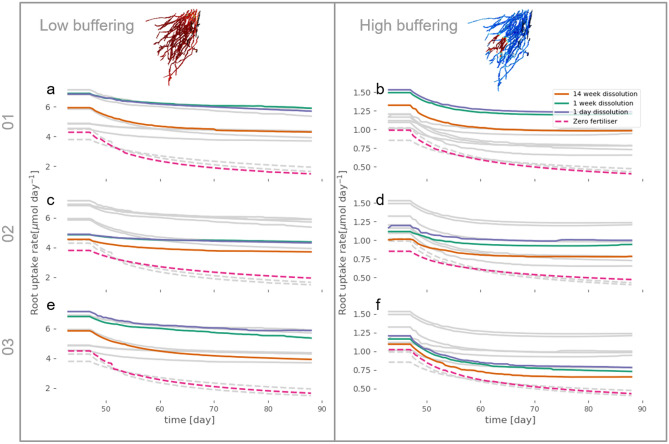
Simulated uptake rate dynamics from 6 weeks until 14 weeks for both the low and high buffering soil. Root uptake was started at 6 weeks following dissolution of the fertiliser from day zero. Each soil has the same total quantity of P initially. The first column shows the low buffering case (buffer power 40) and the second shows the high buffering case (buffer power 519). Each row highlights one of the three root systems showing the 4 fertiliser dissolution cases: Zero fertiliser, 14-week dissolution, 1-week dissolution and 1-day dissolution within that soil type. (**a**) Low buffering soil with 01 root system. (**b**) High buffering soil with sample 01 root system. (**c**) Low buffering soil with sample 02 root system. (**d**) High buffering soil with sample 02 root system. (**e**) Low buffering soil with sample 03 root system. (**f**) High buffering soil with sample 03 root system. For visualisation purposes, root images show uptake rate at 43 days. The faded lines show the other root system measurements in each soil type to ease comparison between plants.

**Figure 6:**
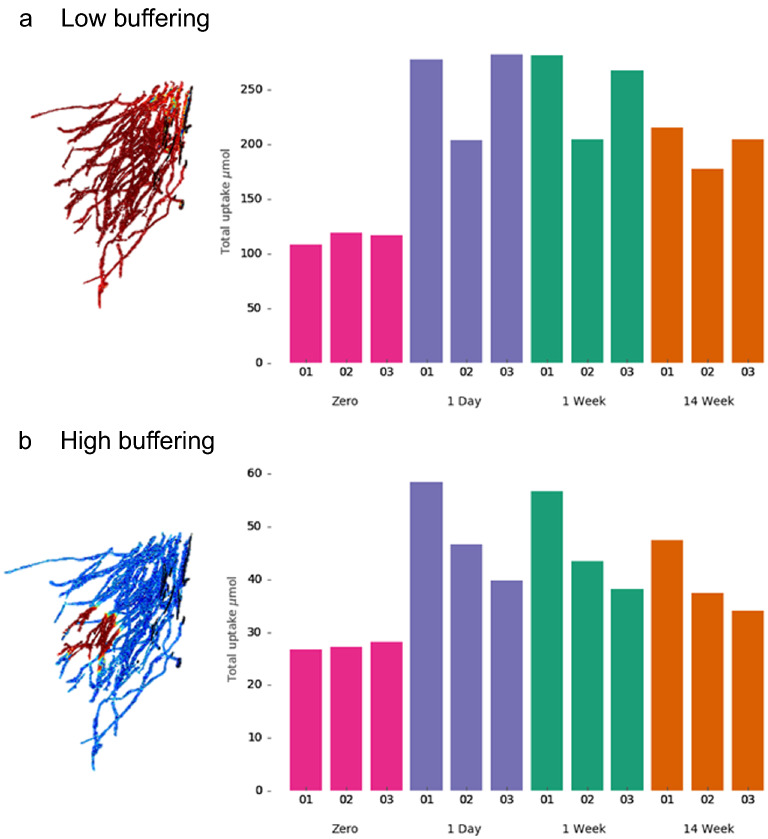
Simulated total P uptake integrated over the whole experiment in (**a**) low buffering soil (**b**) high buffering soil. Different cases represent P dissolving from a pellet over 1-day, 1-week, and 14-weeks, and a case with no added P fertiliser (zero fertiliser) for three different root systems (samples 01, 02, 03). For visualisation purposes, root images show uptake rate at 43 days.

Averaged values reported in Fig. 4 obscure some consistent differences between the treatments and replicates that can be detected using the combined modelling and imaging approach. When comparing P uptake rate dynamics of 1-week and 1-day dissolution with 14-week dissolution, we found faster dissolution led to greater absolute plant P uptake in both soils in all replicates (Fig. 5). However, 1-day dissolution only obtained a slightly higher uptake rate than 1-week in both soils (Fig. 5). Sample 02 had the steadiest uptake rate (Fig. 5c,d) due to having the most evenly distributed roots within the soil, as indicated by mean soil–root distances in Table 1. This may explain why sample 02 has the highest total uptake in the zero fertiliser case in the low buffering soil despite it having the lowest root surface area (Fig. 6a). Unlike the more densely packed roots, the roots of sample 02 have explored more soil and are not competing for the same soil P supply, thus have access to more of the total P. This effect is diminished in the high buffering soil since the depletion zones around roots are smaller in these low mobility zones and thus less likely to overlap with neighbouring roots (Fig. 6b).

When comparing between soils with different buffer powers, we found that absolute P uptake was almost an order of magnitude lower in the high buffering soil compared with the low buffering soil. This is consistent with previous knowledge regarding soil sorption capacity^37^, however, the relationship between buffer power and plant P uptake is not a linear transformation since the root uptake is non-linear.

### Total uptake and efficiency per replicate

When comparing the total P uptake between replicates, the uptake for sample 02 was lower than the uptake for sample 03 in each fertilised treatment in low buffering soil (Fig. 6a). However, in high buffering soil, the opposite was true. Sample 02 had greater P uptake than sample 03 in all fertilised cases. Samples 01 and 02 showed a similar decrease in uptake when changing from high to low buffering, but sample 03 performed noticeably worse. Sample 03 has a high surface area, but the majority of its root length density is concentrated further away from the P source compared to the other two replicates (no roots are within 1.8 mm of the pellet, Table 1 and see Fig. 2c). Hence, when P mobility is reduced in the high buffering soil, much of sample 03’s root system has limited access to P from the pellet, which explains its poor relative performance in the high buffering soil.

**Figure 7:**
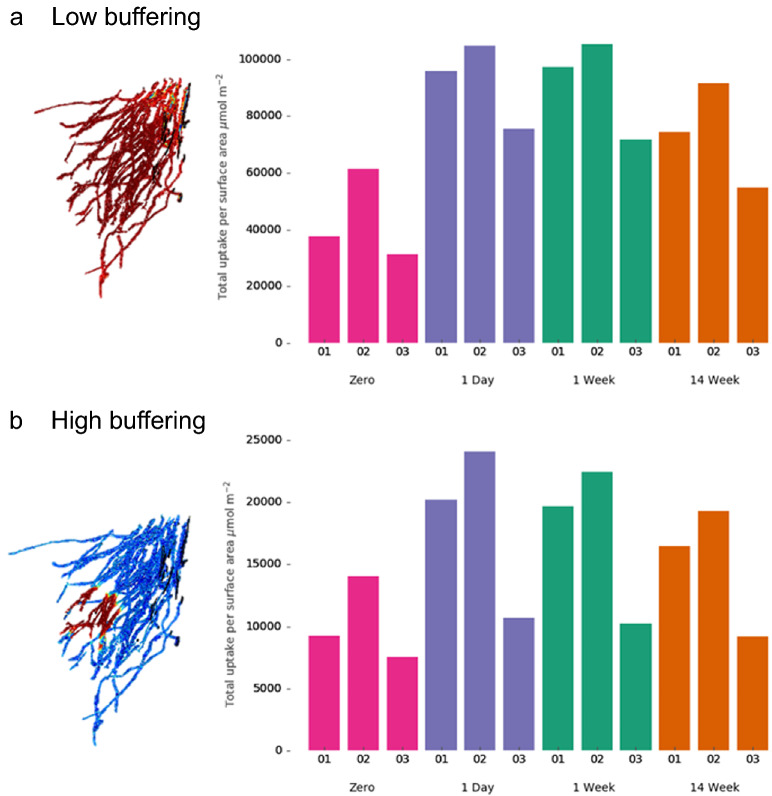
Simulated total P uptake per root surface area integrated over the whole experiment in (**a**) low buffering soil (**b**) high buffering soil. Different cases represent P dissolving from a pellet over 1-day, 1-week, and 14 weeks, and a case with no added P fertiliser (zero fertiliser) for three different root systems (samples 01, 02, 03). For visualisation purposes, root images show uptake rate at 43 days.

However, different patterns emerge when we consider P uptake per root surface area, a measure of P uptake efficiency relative to plant carbon investment in its root system (Fig. 7). In terms of P uptake per root surface area, sample 02 is the most efficient root system for both soil types (it takes up the most P per root surface area), because it has the smallest surface area, but explores the most soil (Table 1). Due to its lower surface area, each root is competing less with other roots for P. This is particularly evident in the zero fertiliser case, compared with the denser root systems. On the other hand, sample 03 performs poorly in both soils; particularly in the high buffering soil. This is because its dense root system means P is quickly depleted around the roots, but P mobility is too low for the fertiliser to reach this area due to the roots being further from the P source i.e. they are in the wrong place. Furthermore, the effective diffusion distance is expected to be reduced in the high buffering soil.

## Discussion

Based on the model results, when averaged across the different root systems, P uptake was similar between the different dissolution rates for the pellet. In fact, the only difference between the mean results is that P fertilisation is expected to lead to higher P uptake, which is perhaps an obvious result, but at least demonstrates that the fertiliser from the “pellet” is reaching the plant roots and affecting overall uptake. However, the major advantage of the image-based modelling approach used here is the ability to more thoroughly explore the variability over time and between different root systems, effectively allowing us to disentangle the sources of variation between replicates. This would not be possible using one dimensional models where root length density is a function of depth since these functions would be very similar between replicates in this study, see Fig. 2b. Faster dissolution rates had consistently higher uptake rates (Fig. 5), which would suggest that the rapid dissolution of fertilisers such as TSP^11^ would lead to higher P uptake compared with the slow dissolution of struvite^12^. This result helps explain the processes behind the results of Nkebiwe et al.^15^ who found that mobile fertiliser sources had greater yield and nutrient uptake compared to less mobile sources of fertiliser. Talboys et al.^9^ found that fertiliser recovery was similar between TSP (a relatively soluble source) and struvite for spring wheat at harvest, but that wheat plants fertilised with struvite developed significantly fewer mature grain heads than those fertilised with TSP. As with our model, they also found that early uptake of P was significantly reduced (39%) in the struvite despite total uptake being similar, and this may be the reason for differences in grain development. Talboys et al.^9^ therefore argue for a combination of slow-release and fast-release fertilisers to ensure the needs of the plant at different times are being met. Our model could be further developed to identify the optimal combinations of the two types of fertiliser to provide appropriate levels of P to the plant.

Root system architecture and the distance between the pellet and the roots play a more prevalent role in high buffering soils, as the active region of root uptake and uptake variability is higher under these buffering conditions (Fig. 6). However, our results also demonstrate that the optimal root architecture may differ between different soil buffering conditions, as sample 03 had high total uptake in the low buffering soil but the lowest total P uptake of the replicates in the low buffering soil (Fig. 6). This was because sample 03 had a high root surface area, allowing uptake over a large surface, but the roots were more clumped and further away from the pellet so when diffusion was more limited (high buffering) the depleted regions near the dense root were not replenished by the fertiliser.

From our findings, root system architecture seems to be important for uptake efficiency (P uptake per root surface), more so than for total uptake. However, the traits that are most beneficial to uptake evolve over time, which could be easily overlooked on shorter term simulations^8^. In the zero fertiliser case, the root system with the highest surface area had the highest initial uptake rate, (Fig. 5) sample 03, which is in agreement with previous studies^22^. However, it was the exploration extent (here measured using mean soil–root distance, Table 1) that was most important for long term total P uptake. Although sample 02 starts with a lower uptake rate due to its lower surface area (Fig. 5), it has the highest total uptake over the whole period since it explores more of the soil (lower mean soil–root distances, Table 1) (Fig. 6a). Which of these strategies (high root surface area vs high soil exploration) is the most effective depends on which measure is most important. Results highlight a benefit for a sparsely distributed rooting system when there is little P more uniformly distributed throughout the soil. However, given high uptake in early growth is very important to crop yields^38^, the higher total uptake may be unable to compensate for reduced uptake early on. Single time point measurements of root length density or short-term simulations would not be able to capture these nuances that affect P uptake.

In addition, the root traits that are beneficial for uptake efficiency (greater exploration) may not be beneficial for total P uptake. For example, in the fertilising cases sample 03 has higher total P uptake than sample 02 in the low buffering case but the worst uptake efficiency (Figs. 6 and 7). This is because sample 03 has more roots and, while increasing root volume is a good way to increase P uptake^39^, this should also be balanced with carbon investment^40^. This observation is important because optimal root traits may depend on which factor is yield limiting in a particular agricultural scenario. For example, growing corn in northern Europe may be limited by light but not P availability, thus photosynthate should be spent on above ground organs to maximise yield. In this case, assuming the soil has plenty of available P, high P uptake efficiency is preferable to total P uptake as this will allow greater investment in above ground material, which functions to increase carbon capture and therefore yield^40^. In this scenario, the sample 02 root system which has high uptake efficiency is optimal. On the other hand, where low P availability is the limiting factor rather than light, root systems which have high total P uptake would be preferable and the sample 01 root system is more optimal.

All the comparisons made in this study are between the same species and variety of plant, grown under the same conditions. As such, the study provides better controlled protocols than any field experiment can guarantee. Despite these controls, results highlight large variabilities. Given the variability seen even under these controlled conditions, we expect much greater differences would be observed between different species, varieties and environments. In addition, plants have many different strategies to increase P uptake that are not considered by our present model, including organic-acid exudation, mycorrhizal symbioses and growing fine lateral roots and root hairs^41,42^. The roots we measured from the CT images were only those roots larger than an absolute minimum of 180 µm, due to the limitations of XCT imaging on samples at this scale. Therefore, fine roots and certainly root hairs were missed in these analyses, and these roots are likely to be those that would respond to localised P since they are less costly to produce in terms of carbon^41,42^. The advantages of XCT imaging for maintaining geometric information e.g. about root distribution vs clustering outweigh the disadvantage in losing fine roots for this study, though future work should investigate the role of lateral roots and root hairs further. Soil moisture was assumed constant in the simulation, an assumption that is more relevant to laboratory experiments, where soil moisture is controlled, than to crops in the field. However, P movement is often assumed to be dominated by diffusion due its rapid sorption onto soil particles, and ignoring advection is a common simplification for P models^43^. Although the validity of this assumption for P has been brought into question by the recent study of Petroselli et al.^11^, who found evidence of advection when measuring P concentrations at increasing distance from a fertiliser pellet using microdialysis. However, the results were more likely explained by the advection of fertiliser particulates rather than of P in solution, which is outside the scope of this study. Including soil moisture equations to monitor the advection of P by water flow was not feasible in the current study due to the size of the computational mesh. Ultimately, there was a trade-off between the detail of the root systems and the complexity of mathematical model; we did not have available memory to solve both P advection–diffusion and soil moisture with Richards’ equation while using large and detailed model domains in reasonable time.

In conclusion, our spatially explicit and time-resolved model allowed investigation of the relative roles of root architecture, fertiliser dissolution rate, and soil buffer power in determining P uptake by a plant. More specifically, faster dissolution led to a higher short-term uptake rate, but we found diminishing returns with fertilisers dissolving within one week. In addition, while greater total root length increased total P uptake, smaller, more spread out root systems took up more P per unit root length and therefore may be considered more efficient, and the specific amount of root investment required might depend on variables such as availability of P, and availability of light and water. Monitoring over an extended time period is important since uptake can change through time, though early P uptake is known to be crucial for high yields. Finally, explicitly including the spatial distributions of the root systems highlights that the optimal balance of root exploration and root density is different in different soils with different buffer powers. These results provide the foundation for a predictive modelling approach to understanding uptake from different P fertilisers in different soils. This approach can be used to investigate sources of high variability within single cultivars, which is not identifiable under larger experimental conditions but is important for understanding the underlying processes rather than only average responses. In addition, it could be a highly valuable tool in rapid screening of different fertilisation approaches for specific soil properties, root architectures, and fertiliser properties, complementing and potentially informing more comprehensive field studies.

## Supplementary Information

Supplementary Figures.

## Data Availability

All data supporting this study is openly available from the University of Southampton institutional research repository at https://doi.org/10.5258/SOTON/D1845.
