# Supplementary material for: A 3D image-based modelling approach for understanding spatiotemporal processes in phosphorus fertiliser dissolution, soil buffering and uptake by plant roots
Source: Sci Rep. 2022 Sep 23;12:15891. doi: 10.1038/s41598-022-19047-1 (PMC9508158; doi:10.1038/s41598-022-19047-1)
Supplement: Supplementary file 1 — Supplementary Figures. [file 41598_2022_19047_MOESM1_ESM.docx]

**A 3D image-based modelling approach for understanding spatiotemporal processes in phosphorus fertiliser dissolution, soil buffering and uptake by plant roots**

Authors: K. A. Williams, D. McKay Fletcher, C. Petroselli, S. A. Ruiz, T. Roose.

The following Supporting Information is available for this article:

**Fig. S1** Process of rough segmentation of roots from soil.

**Fig. S2** Final shape-based segmentation of roots from soil in Dragonfly ORS.

**Fig. S3** Producing a cleaned skeleton of the segmented root system.

**Fig. S4** Measuring root length density with distance from a fertiliser pellet.

**Fig. S5** Process for measuring root-soil distances.

**Fig. S6** 3D rendering of each root system (green) and fertiliser (red) in the subsample used for modelling. a) Root system 01, b) Root system 02, c) Root system 03.


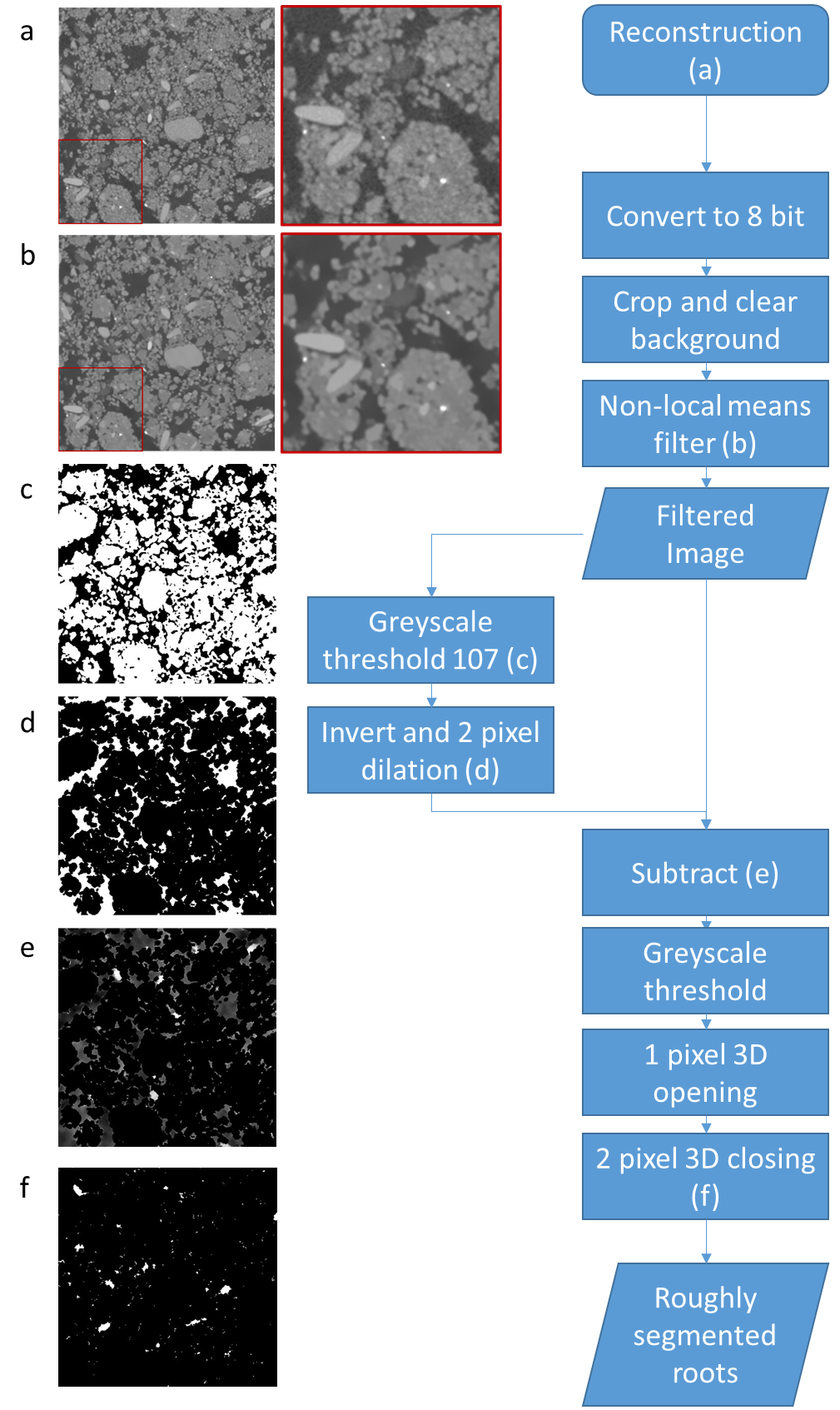


**Fig. S1** Process of segmentation of roots from soil, implemented in ImageJ using the DenoiseEM plugin, and standard tools built into ImageJ. The resulting segmentation contains roots and noise from soil, particularly edges of soil due to partial volume effects.


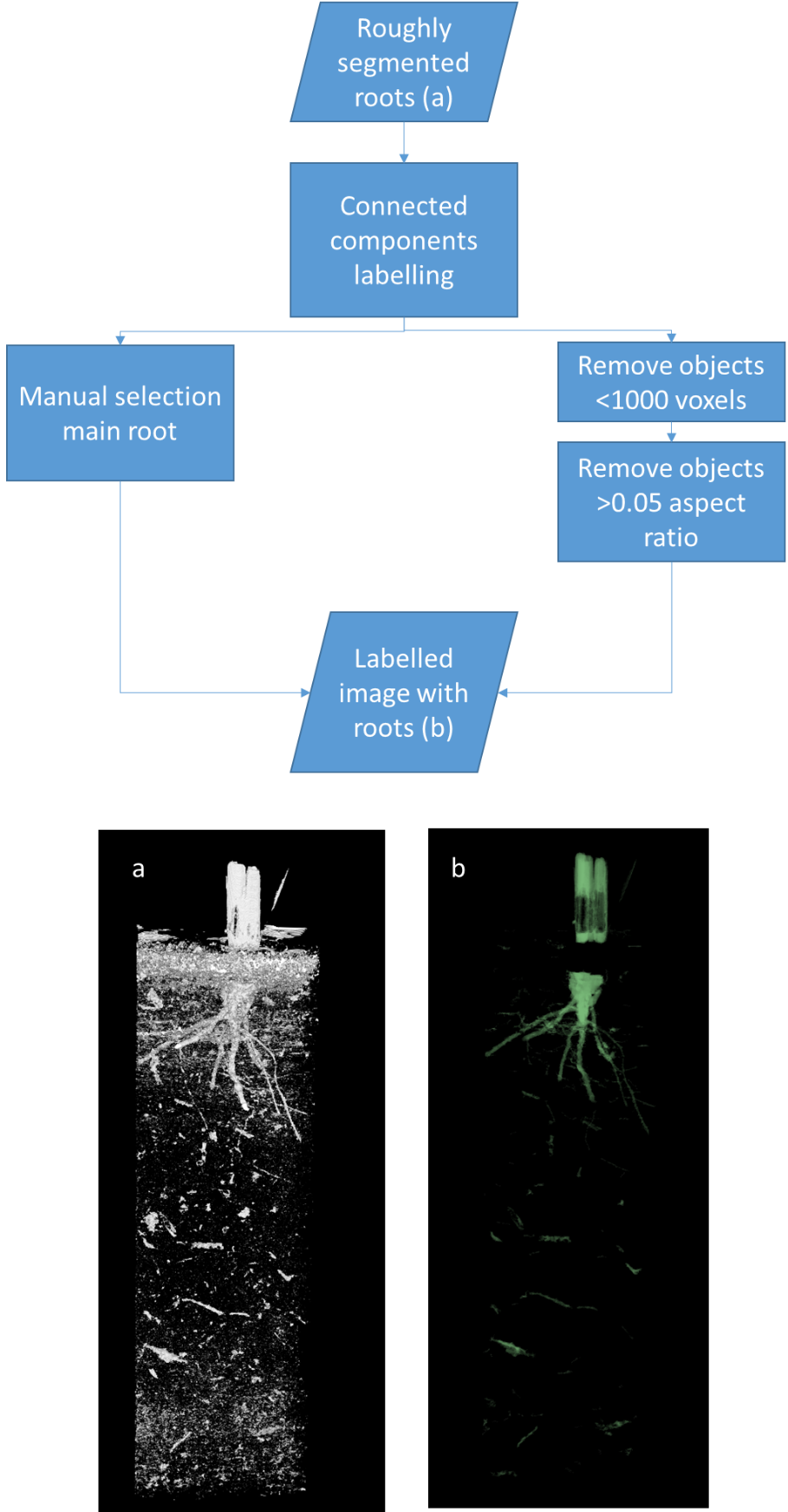


**Fig. S2** Final shape-based segmentation of roots from soil in Dragonfly ORS.


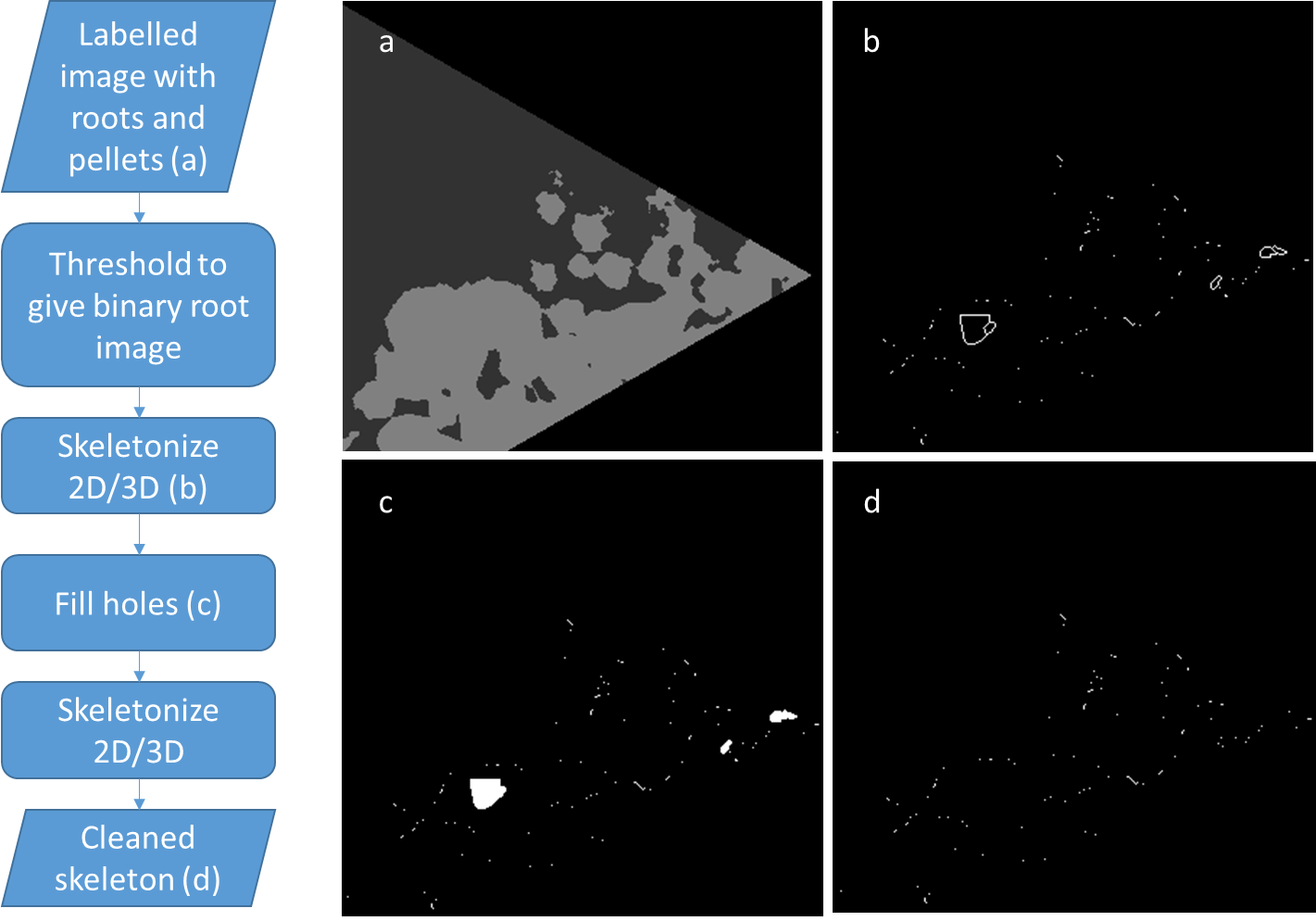


**Fig. S3** Producing a cleaned skeleton of the segmented root system to calculate root length measurements.

**
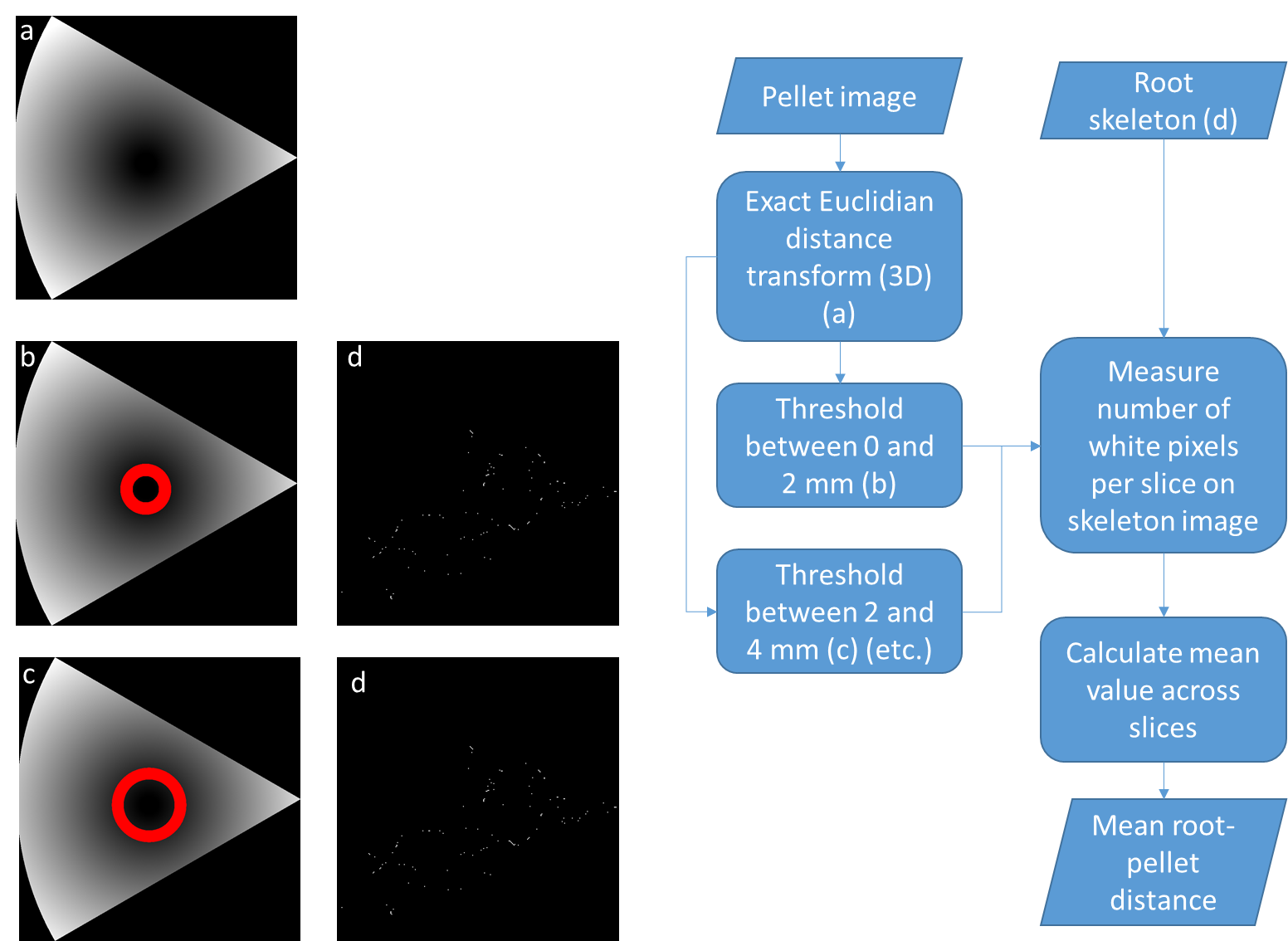
**

**Fig. S4** Measuring root length density with distance from a fertiliser pellet using “redirect to” in ImageJ’s measurement function.

**
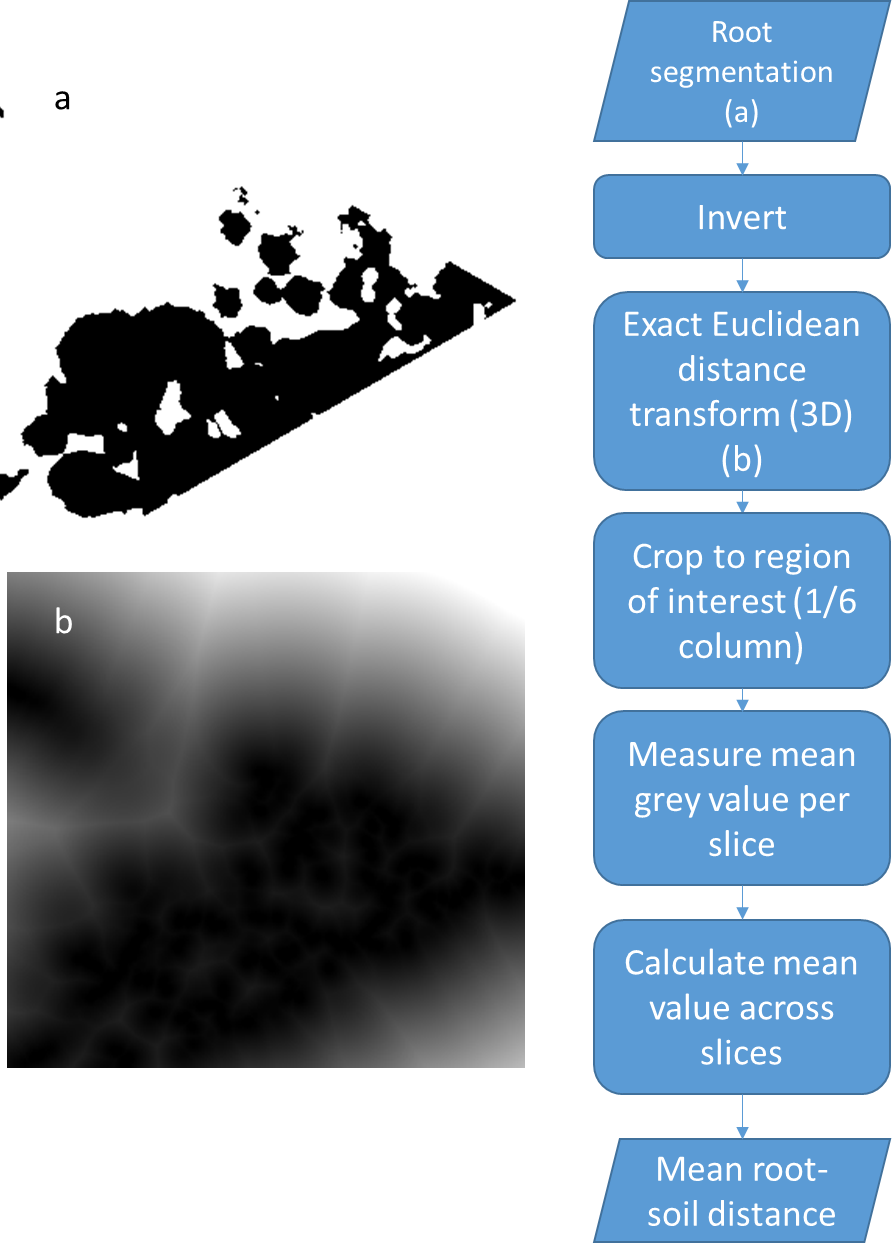
**

**Fig. S5** Process for measuring root-soil distances using an exact Euclidian distance transform from the segmented roots.


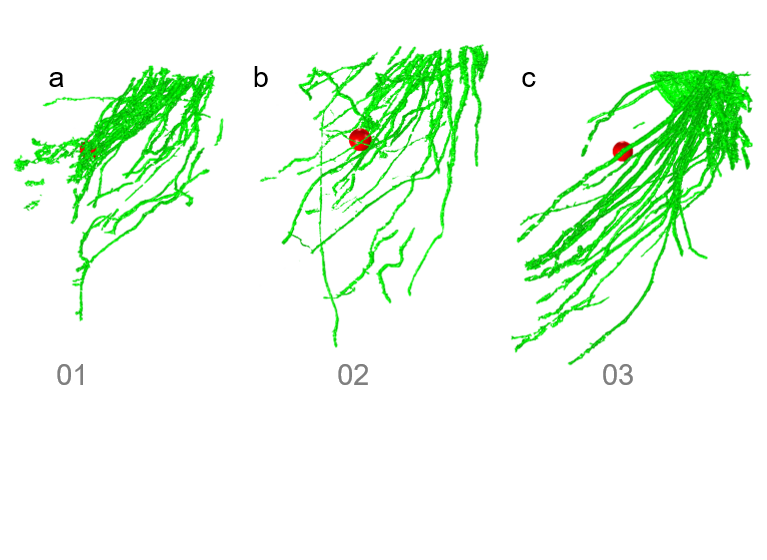


**Fig. S6** 3D rendering of each root system (green) and fertiliser (red) in the subsample used for modelling. a) Root system 01, b) Root system 02, c) Root system 03.
